# Cardiomyocyte and Vascular Smooth Muscle-Independent 11β-Hydroxysteroid Dehydrogenase 1 Amplifies Infarct Expansion, Hypertrophy, and the Development of Heart Failure After Myocardial Infarction in Male Mice

**DOI:** 10.1210/en.2015-1630

**Published:** 2015-10-14

**Authors:** Christopher I. White, Maurits A. Jansen, Kieran McGregor, Katie J. Mylonas, Rachel V. Richardson, Adrian Thomson, Carmel M. Moran, Jonathan R. Seckl, Brian R. Walker, Karen E. Chapman, Gillian A. Gray

**Affiliations:** British Heart Foundation/University Centre for Cardiovascular Science (C.I.W., M.A.J., K.M., K.J.M., R.V.R., C.M.M., J.R.S., B.R.W., K.E.C., G.A.G.), Queens Medical Research Institute, and Edinburgh Preclinical Imaging (M.A.J., A.T., C.M.M.), College of Medicine and Veterinary Medicine, The University of Edinburgh, Edinburgh EH16 4TJ, Scotland, United Kingdom

## Abstract

Global deficiency of 11β-hydroxysteroid dehydrogenase type 1 (11β-HSD1), an enzyme that regenerates glucocorticoids within cells, promotes angiogenesis, and reduces acute infarct expansion after myocardial infarction (MI), suggesting that 11β-HSD1 activity has an adverse influence on wound healing in the heart after MI. The present study investigated whether 11β-HSD1 deficiency could prevent the development of heart failure after MI and examined whether 11β-HSD1 deficiency in cardiomyocytes and vascular smooth muscle cells confers this protection. Male mice with global deficiency in 11β-HSD1, or with *Hsd11b1* disruption in cardiac and vascular smooth muscle (via *SM22*α*-Cre* recombinase), underwent coronary artery ligation for induction of MI. Acute injury was equivalent in all groups. However, by 8 weeks after induction of MI, relative to C57Bl/6 wild type, globally 11β-HSD1-deficient mice had reduced infarct size (34.7 ± 2.1% left ventricle [LV] vs 44.0 ± 3.3% LV, *P* = .02), improved function (ejection fraction, 33.5 ± 2.5% vs 24.7 ± 2.5%, *P* = .03) and reduced ventricular dilation (LV end-diastolic volume, 0.17 ± 0.01 vs 0.21 ± 0.01 mL, *P* = .01). This was accompanied by a reduction in hypertrophy, pulmonary edema, and in the expression of genes encoding atrial natriuretic peptide and β-myosin heavy chain. None of these outcomes, nor promotion of periinfarct angiogenesis during infarct repair, were recapitulated when 11β-HSD1 deficiency was restricted to cardiac and vascular smooth muscle. 11β-HSD1 expressed in cells other than cardiomyocytes or vascular smooth muscle limits angiogenesis and promotes infarct expansion with adverse ventricular remodeling after MI. Early pharmacological inhibition of 11β-HSD1 may offer a new therapeutic approach to prevent heart failure associated with ischemic heart disease.

Interventions to restore perfusion after myocardial infarction (MI) have significantly enhanced acute survival (1). However, many patients survive with injury to their myocardium that is replaced during wound healing by noncontractile scar tissue. In the longer term, structural, functional, and metabolic remodeling of the remaining ventricle to compensate for contractile deficiency and alteration in wall stress promotes progression to heart failure (2). Retention of functional cardiomyocytes is critical in limiting subsequent adverse ventricular remodeling. In experimental models cardiomyocyte loss can be reduced by intervention at the time of reperfusion, but successful translation of these interventions to the clinic has been limited (3, 4). Cardiomyocyte death also occurs in the periinfarct area during infarct healing and scar formation, leading to infarct expansion. Promotion of angiogenesis during this phase can limit infarct expansion and subsequent adverse remodeling (5–9).

Glucocorticoids (physiological cortisol and corticosterone, as well as synthetic forms) are known to suppress angiogenesis (10). Plasma levels of cortisol increase in the hours after MI after activation of the hypothalamic-pituitary-adrenal axis and may protect cardiomyocytes from acute ischemic injury (11–13), but as circulating levels are reduced within days of MI, they are unlikely to impact on the later angiogenesis that is associated with infarct healing. However, active glucocorticoids can also be regenerated locally from circulating inert 11-keto metabolites by the enzyme, 11β-hydroxysteroid dehydrogenase type 1 (11β-HSD1), that is expressed in many cells, including in cardiomyocytes, fibroblasts, and smooth muscle cells in the heart (14). Genetic disruption of *Hsd11b1*, the gene encoding 11β-HSD1, does not change initial ischemic injury in the murine heart after experimental MI but leads to enhancement of periinfarct vessel density (8, 10). Importantly, vessels formed during infarct healing mature to maintain blood flow to the periinfarct area in 11β-HSD1-deficient mice and increased vessel density is associated with shorter and thicker infarcts and with retention of cardiac function (8). Pharmacological inhibitors of 11β-HSD1 have reached phase 2 clinical development for use in diabetes and cognitive dysfunction and have potential as a new therapeutic approach for promotion of angiogenesis, including post-MI (15, 16). However, whether 11β-HSD1 deficiency can prevent maladaptive ventricular remodeling and progression to heart failure after MI has not been examined.

11β-HSD1 activity is not detectable in endothelial cells, but the enzyme is present in the vessel wall where expression and activity is associated with smooth muscle cells (17, 18). Whether glucocorticoids regenerated by 11β-HSD1 in vascular smooth muscle cells act as an endogenous brake upon angiogenesis in the healing infarct, or indeed upon any aspect of ventricular remodeling after MI, is unknown. A recent study has shown that myocardial expression of 11β-HSD1 is increased in an MI-independent model of pathological hypertrophy and that ventricular remodeling can be reversed by pharmacological inhibition of 11β-HSD1 without any change in vessel density (19). This suggests that 11β-HSD1 has effects in the heart over and above those involving angiogenesis, potentially via 11β-HSD1 expressed in cardiomyocytes.

The present study tested the hypothesis that long-term maladaptive structural and functional myocardial remodeling and the development of heart failure after MI are attenuated by 11β-HSD1 deficiency. To establish the contribution of 11β-HSD1 specifically in vascular smooth muscle cells and cardiomyocytes to the beneficial outcome after MI seen in mice lacking the enzyme globally, targeted deletion of 11β-HSD1 in these cell types was investigated.

## Materials and Methods

### Experimental animals

All experiments involving animals were approved by The University of Edinburgh Animal Welfare and Ethical Review Body and by the Unite Kingdom Home Office. Experiments used adult male (10–14 wk of age) mice with global deficiency on a C57Bl/6 genetic background (*Hsd11b1*^−/−^) (8, 20), with C57Bl/6 controls, or mice in which deletion was targeted to vascular smooth muscle and cardiomyocytes (21, 22). These *Hsd11b1^fl/fl^Sm22*α*-Cre*^+^ (*Hsd11b1*^CVCre+^) were generated by crossing *Sm22*α*-Cre* mice with *Hsd11b1^fl/fl^* mice, homozygous for a “floxed” allele of *Hsd11b1* (generated by Artemis Pharmaceuticals directly onto a C57BL/6J background). LoxP sites were placed flanking exon 3 of the mouse *Hsd11b1* gene, excision of which results in a “null allele” by “out of frame splicing” from exon 2 to exon. Controls were *Hsd11b1^fl/fl^* (*Cre*−) littermates (*Hsd11b1*^CVCre−^). After *Cre* genotyping, appropriate targeting was confirmed by determination of *Hsd11b1* RNA and 11β-HSD1 protein content in myocardium, aorta, liver, and skeletal muscle.

### Induction of MI

MI was induced by coronary artery ligation (CAL), as previously described (8, 23), in *Hsd11b1*^−/−^ mice, in age matched C57Bl/6 mice (wild type [WT]), in *Hsd11b1*^CVCre+^, and in *Hsd11b1*^CVCre−^ mice. Briefly, after injection of analgesic (buprenorphine, 0.05 mg/kg, sc) and intubation for mechanical ventilation (120 strokes/min, 200-μL stroke volume; HSE-Harvard MiniVent), the chest was opened at the fourth intercostal space, and the pericardium was removed to allow ligation of the left anterior descending coronary artery (6-0 PROLENE suture; Ethicon). Successful ligation was confirmed by blanching of the left ventricle (LV), and the chest was closed using a 5-0 MERSILK suture (Ethicon), ensuring that no air remained in the chest cavity. The skin was closed using 9-mm stainless steel autoclips (Harvard Apparatus). After surgery, 1.5-mL sterile saline (0.9%, sc) was administered to aid recovery. Twenty-four hours after induction of MI, a blood sample (45 μL) was collected from the tail vein into a tube containing sodium citrate buffer for assay of troponin I by ELISA (Life Diagnostics High Sensitivity Mouse Cardiac Troponin-I ELISA kit) to assess the extent of injury (24).

### Structural and functional characterization

Eight weeks after CAL surgery, cardiac structure, function, and infarct size was measured using magnetic resonance imaging (MRI), as previously described (25). Temperature was maintained at 37°C, respiration rate at 50–60 breaths per minute, and heart rate at 500–550 beats per minute during imaging in isoflurane (1.3%–1.8%) anesthetized mice. Animals were placed in a supine position inside a 7T MRI scanner (Agilent Technologies) with a 39-mm quadrature radiofrequency coil. Short axis cardiac images were acquired using an electrocardiogram (ECG) triggered and respiratory-gated gradient echo “cine” sequence (repetition time/echo time = 7.3/2.7 ms with a flip angle of 15°) with gradient and radiofrequency spoiling. Nine consecutive 1-mm-thick slices from 12–13 time frames were acquired, which encompassed the entire heart from base to apex. The field of view was 30 mm with a 192 × 192 matrix, and 4 averages were used. ImageJ software (National Institutes of Health) was used to assess LV end-diastolic volume, LV end-systolic volume, and ejection fraction (EF). Infarct size was measured by segmenting each short axis image into 20 sections. Any section which was thinned and akinetic or dyskinetic over the cardiac cycle was designated as infarcted tissue. Gray scale contrast also allowed visual confirmation of infarcted tissue. Infarct length was quantified by adding together the endo- and epicardial circumference of infarcted tissue and dividing this by the sum of the total endo- and epicardial circumferences.

After MRI, mice were killed by cervical dislocation, and the lungs collected and weighed to assess pulmonary edema. Hearts were weighed and either frozen at −80°C for subsequent RNA extraction and analysis, or processed and embedded in paraffin wax for histological and immunohistochemical analysis.

In a separate study, *Hsd11b1*^CVCre+^ and *Hsd11b1*^CVCre−^ mice underwent CAL for induction of MI as above, or sham operation. Structure and function were investigated by high-resolution ultrasound (Vevo 770 High Resolution Ultrasound Scanner; Visualsonics) 7 days after surgery. Briefly, long axis views of the heart in ECG-Gated Kilohertz Visualization mode and M-mode were acquired in isoflurane (2%) anesthetized mice placed on a heated table to maintain body temperature at 37°C, heart rate was maintained in the range of 500–550 beats per minute. Ventricular area at end systole (LV end-systolic area) and at end diastole (LV end-diastolic area) were collected from ECG-Gated Kilohertz Visualization traces using Vevo Image Analysis Software (Visualsonics), and this permitted calculation of EF. Fractional shortening was calculated from M-mode. In this study, hearts were perfusion fixed in situ and prepared for histological assessment of infarct area, vessel density, and macrophage content.

### Molecular analysis

RNA was extracted from homogenized tissue using a TRIzol Plus RNA Purification kit (Invitrogen) according to the manufacturers' instructions. After confirmation of RNA concentration and the A_260_/A_280_ ratio (Nanodrop 1000 version 3.3; Thermo Scientific), each sample was deoxyribonuclease I treated to remove genomic DNA. RNA was reverse transcribed (High Capacity cDNA Reverse Transcription kit; Applied Biosystems), and cDNA synthesis was performed in a thermal cycler using the next conditions: 25°C for 10 minutes, 48°C for 40 minutes, and 95°C for 5 minutes, then cooled to 4°C. *Hsd11b1* expression was assessed for each sample in triplicate using a TaqMan Gene Expression Assay (Mm00476182_m1). For cardiac genes quantitative real-time PCR (qRT-PCR) was performed to assess mRNA levels of atrial natriuretic peptide (ANP) (*Nppa*), collagen 1α2 (*Col1a2*), collagen 3α1 (*Col3a1*), TGFβ (*Tgfb1*), α-myosin heavy chain (α-MHC) (*Myh6*), and β-MHC (*Myh7*) using a Lightcycler 480 (Roche) and the TaqMan Fast Advanced template or the Roche Fam Hydrolysis (Roche) template as appropriate. The internal control for all qRT-PCR experiments was β-actin (*Actb*), which was similar in all groups. Primers (Supplemental Table 1) were designed in house and prepared by Invitrogen. A standard curve was prepared by pooling 2 μL from each cDNA sample and making serial dilutions in ribonuclease -free water.

### Western blotting

11β-HSD1 protein content was assessed by Western blotting as previously described (26), in the heart, liver, aorta, and skeletal muscle to confirm appropriate deletion in *Hsd11b1*^CVCre+^ mice. Briefly, 50 mg of tissue were homogenized in 300-μL lysis buffer (radioimmunoprecipitation assay [RIPA] buffer; Sigma) and protease and phosphatase inhibitor cocktails (Sigma), then centrifuged at 12 000*g* for 15 minutes at 4°C. Proteins were separated by SDS-PAGE and then transferred (Trans-Blot Semi Dry Transfer Cell; Bio-Rad) to a nitrocellulose membrane. After blocking (milk powder in Tris-buffered saline buffer with Tween 20), the membrane was incubated overnight at 4°C with primary 11β-HSD1 antibody (1:10 000 in 5% BSA, generated in house in sheep) (26). The membrane was washed before application of horseradish peroxidase-conjugated donkey-antisheep secondary antibody (1:5000, ab97125; Abcam) and then washed again and exposed to antimouse β-actin (1:10 000; Cell Signaling Technology) as a loading control, to horseradish peroxidase-conjugated rabbit antimouse secondary antibody (ab6728; Abcam), then developed (ECL Prime Western Blotting Detection Reagent; GE Healthcare) and exposed to x-ray film for detection. 11β-HSD1 protein levels was calculated by normalizing the density of this band to that of β-actin protein (see Table 1).

**Table 1.** Antibody Table

Peptide/Protein Target	Name of Antibody	Manufacturer, Catalog Number, and/or Name of Individual Providing the Antibody	Species Raised in; Monoclonal or Polyclonal	Dilution Used
Mac 2 (galectin-3)	Antimouse/human Mac 2	Cedarlane, CL8942AP	Rat; monoclonal	1:6000
Ym-1 (ECF-L)	Ym-1 antibody	STEMCELL Technologies, 01404	Rabbit; polyclonal	1:500
CD 31 (Pecam)	Anti-CD-31 antibody	Abcam, ab28364	Rabbit; polyclonal	1:50
Isolectin B4	Isolectin GS-IB4 from *Griffonia simplifolica*	Thermo Fisher, I21413	Rabbit	1:100
Wheat germ agglutinin	Rhodamine-labelled wheat germ agglutinin	Vector Laboratories, RL-1022	Goat	1:75
β-Actin	β-Actin antibody	Cell Signaling Technology, 4967S	Rabbit; polyclonal	1:1000
11β-HSD1	11β-HSD1	Professor Karen Chapman	Sheep	1:1000

### Histological and immunohistochemical analysis

Infarct area and thickness were assessed in Masson's trichrome-stained paraffin sections. The infarcted area was selected manually and expressed as a percentage of the total area of the LV (Image Pro 6.2 and a Stereologer Analyzer 6; MediaCybernetics). Infarct thickness was determined by averaging scar thickness from 5 equally spaced points along the length of the infarct. For macrophages, slides were incubated overnight with biotinylated rat antimouse Mac 2 (Galectin-3; a 30-kDa carbohydrate-binding protein expressed on the surface of macrophages) (1:6000; Cedarlane), or for alternatively activated macrophages with rabbit antimouse Ym-1 (chitinase-like 3; a marker highly expressed in alternatively activated macrophages) (1:500; STEMCELL Technologies) followed by a biotinylated goat antirabbit (Vector). Color was developed with a few drops of diaminobenzidine (DAB) (Vector) according to the manufacturer's instructions. A color threshold was set manually so as to only select positive brown staining for Mac 2 and the area of this positive staining within the infarct was calculated automatically. Total macrophage infiltration was then expressed as the percentage area of positive brown DAB staining within the infarct. Periinfarct angiogenesis was assessed in tissue sections by detection of immunoreactive CD31 (rabbit antimouse CD31, 1:50; Abcam) in PBS with a biotinylated goat antirabbit (Vector) secondary antibody. DAB solution was added to each section for visualization. Angiogenesis was assessed in 20 randomly assigned fields of view around the infarct border. The number of CD31^+^ vessels were measured and categorized according to size; capillaries (<4 μm in diameter), small arterioles (4–200 μm in diameter), and large arterioles (>200 μm in diameter).

Cardiomyocyte cross-sectional area was assessed in isolectin B4 (Alexa Fluor-conjugated isolectin B4, 1:100; Thermo Fisher) and wheat germ agglutinin (wheat germ agglutinin-rhodamine, 1:75; Vector)-stained paraffin sections as previously described (27). Five randomly selected regions of interest were visualized in the LV-free wall. Adobe Photoshop CS5 Extended (Adobe) was used to calculate cross-sectional area in cardiomyocytes, which appeared to be cut in the short axis as judged by 1) a nucleus in the middle of the cell and 2) surrounded by capillaries which were also cut in the short axis.

### Statistical analysis

Prism 6f for Mac OS X (GraphPad Software, Inc) was used for two-way ANOVA (7-d *Hsd11b1*^CVCre+^ and *Hsd11b1*^CVCre−^, sham and MI) or 2-tailed unpaired Student's *t* tests (comparisons of *Hsd11b1*^−/−^ and WT, or *Hsd11b1*^CVCre+^ and *Hsd11b1*^CVCre−^). All values are expressed as mean ± SEM, statistical significance was accepted at *P* < .05.

## Results

### Global, but not cardiomyocyte and vascular smooth muscle cell, disruption of *Hsd11b1* prevents infarct thinning and expansion during the development of heart failure

**Figure 1.**
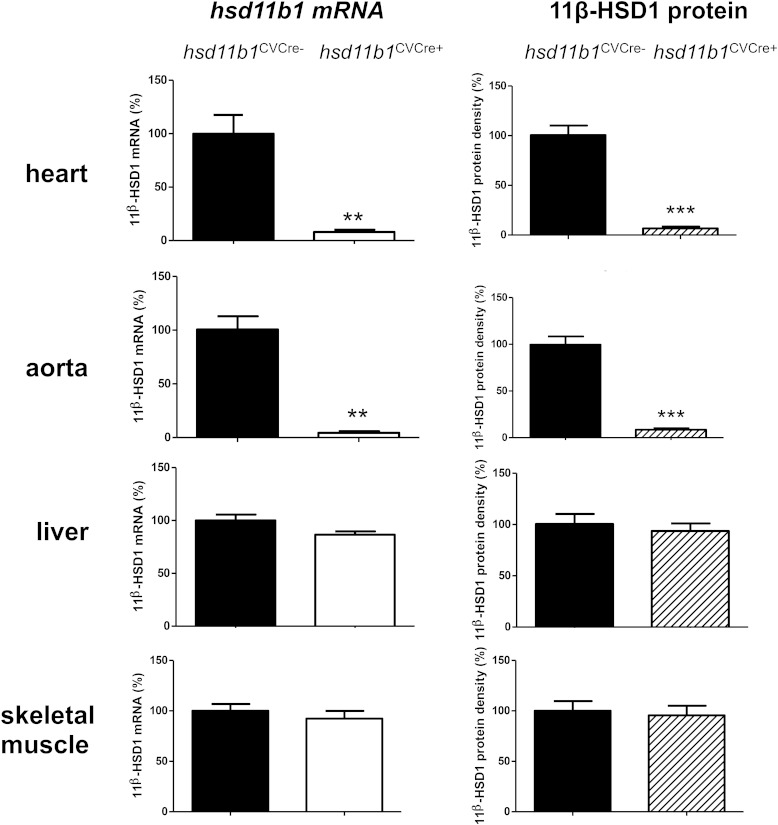
Confirmation of cardiovascular-specific 11β-HSD1 deletion. *Hsd11b1* mRNA and 11β-HSD1 protein were assessed by real-time PCR (normalized to β-actin housekeeping gene) and Western blotting (normalized to β-actin loading control), respectively, in heart, aorta, liver, and skeletal muscle from *Hsd11b1^fl/fl^Sm22*α*-Cre*^+^ (*Hsd11b1*^CVCre+^) and control *Hsd11b1^fl/fl^Sm22*α*-Cre*^−^ (*Hsd11b1*^CVCre−^) mice. Expression in *hsd11b1*^CVCre+^ is expressed relative to maximum achieved in tissue from *Hsd11b1*^CVCre−^; ***, *P* < .001; **, *P* < .01 vs parallel control; n = 4/group.

**Figure 2.**
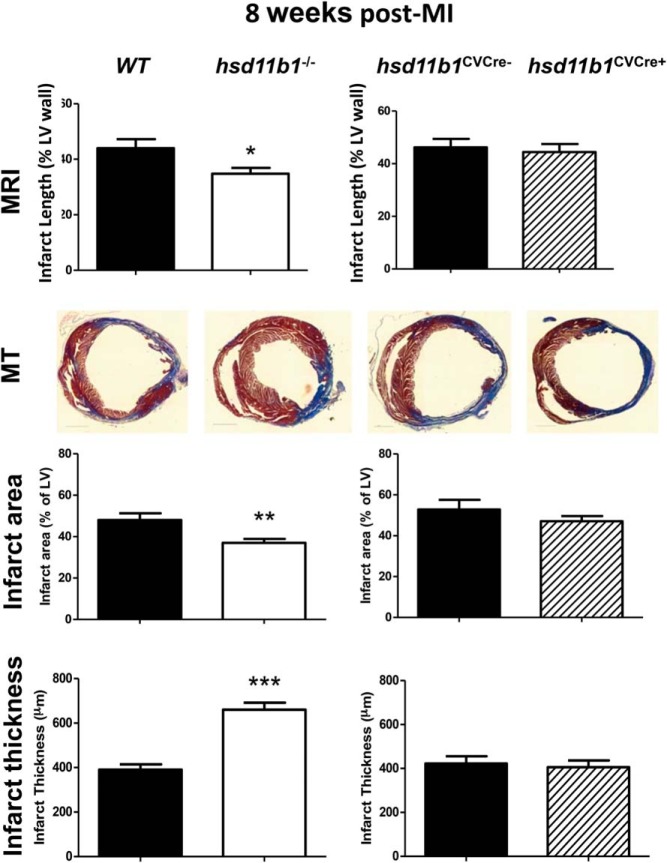
Global, but not cardiovascular-specific, 11β-HSD1 deficiency reduces infarct expansion after MI. Infarct injury was compared in WT and global 11β-HSD1-deficient mice (*Hsd11b1*^−/−^), and in mice with cardiomyocyte and vascular smooth muscle-specific deletion (*Hsd11b1*^CVCre+^) and respective control (*Hsd11b1*^CVCre−^) at 8 weeks after induction of MI by (top panel) MRI and by (lower panels) histology (Masson's trichrome, MT) to determine infarct area and infarct thickness. Paired groups were compared by Student's *t* test; *, *P* < .05; **, *P* < .01; ***, *P* < .005; n = 6–8/group.

*Hsd11b1* mRNA and 11β-HSD1 protein levels were both significantly reduced in the myocardium and aorta, but not in liver or skeletal muscle, of *Hsd11b1*^CVCre+^ mice relative to their littermate controls (*Hsd11b1*^CVCre−^), consistent with selective disruption of gene expression in cardiomyocytes and vascular smooth muscle directed by *SM22-*α-*Cre* (Figure 1) (21). Blood pressure, contractile function and heart weight to body weight ratio were not significantly different to WT mice (Supplemental Figure 1). Troponin I in plasma collected from the tail vein 24 hours after CAL, a measure of ischemic injury, increased to over 40 ng/mL in all groups (Supplemental Figure 2), confirming that the extent of the initiating myocardial injury is not influenced by 11β-HSD1 deficiency (8). In contrast, when injury was assessed at 8 weeks after MI (Figure 2), either in vivo by MRI or in histological sections, infarct area was significantly less (*P* < .05), and average thickness was significantly greater (*P* < .005) in mice with global 11β-HSD1 deficiency compared with their WT controls. This attenuation of infarct expansion was not seen in *Hsd11b1*^CVCre+^ relative to their *Hsd11b1*^CVCre−^ littermate controls (Figure 2C).

### Ventricular dilation is reduced and function improved in mice with global, but not cardiomyocyte and vascular smooth muscle cell, depletion of *Hsd11b1*

**Figure 3.**
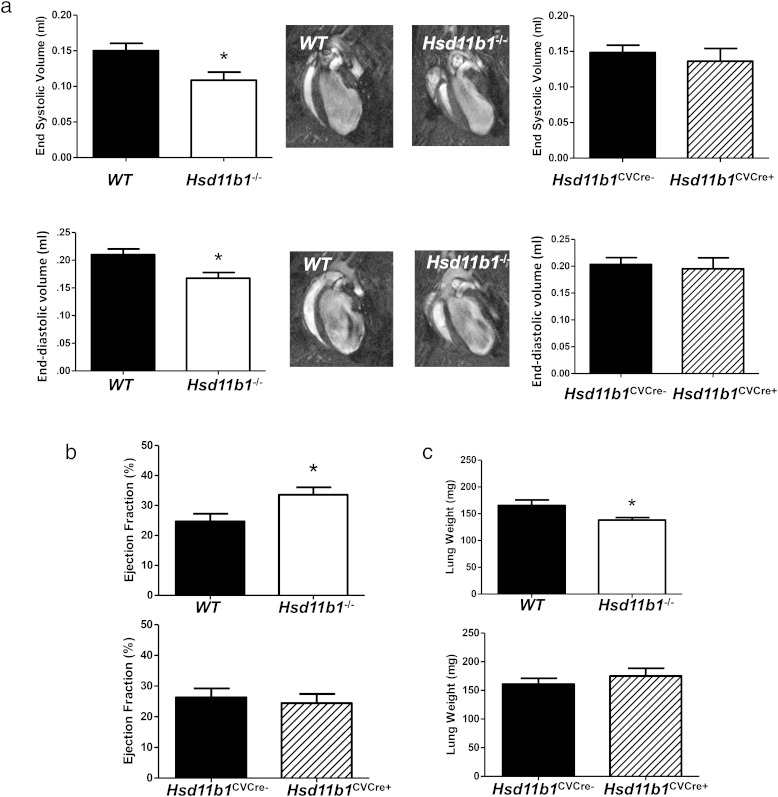
Global, but not cardiovascular, 11β-HSD1 deficiency reduces LV dilatation, loss of contractile function, and pulmonary edema after MI. Structural (A) and functional (B) remodeling was compared by MRI in WT and global 11β-HSD1-deficient mice (*hsd11b1*^−/−^) and in mice with cardiomyocyte and vascular smooth muscle-specific deletion (*hsd11b1*^CVCre+^) and respective floxed control (*hsd11b1*^CVCre−^) at 8 weeks after induction of MI by CAL. The middle panels show 4 chamber views collected by cine-MRI at end-systole (top) and end-diastole (bottom). Lungs were collected from mice after imaging and weighed to detect pulmonary edema (C). Paired groups were compared by Student's *t* test; *, *P* < .05; **, *P* < .01; ***, *P* < .005; n = 6–8/group.

Analysis of structural and functional remodeling by MRI 8 weeks after induction of MI revealed a significant reduction in LV end-diastolic and end-systolic volumes in mice with global, but not tissue-specific, depletion of 11β-HSD1 (*P* < .05) (Figure 3A). EF was increased by 41 ± 9% in *Hsd11b1*^−/−^ mice (*P* < .05) (Figure 3B), consistent with greater retention of contractile function in this group only. Lungs collected from *Hsd11b1*^−/−^ mice 8 weeks after induction of MI were also significantly lighter (*P* < .05) (Figure 3C) than WT mice or mice with tissue-specific deletion, suggesting that *Hsd11b1*^−/−^ mice have reduced tendency to develop pulmonary edema.

### Global, but not cardiomyocyte and vascular smooth muscle cell, depletion of *Hsd11b1*^−/−^ reduces cardiac hypertrophy and expression of fetal genes

**Figure 4.**
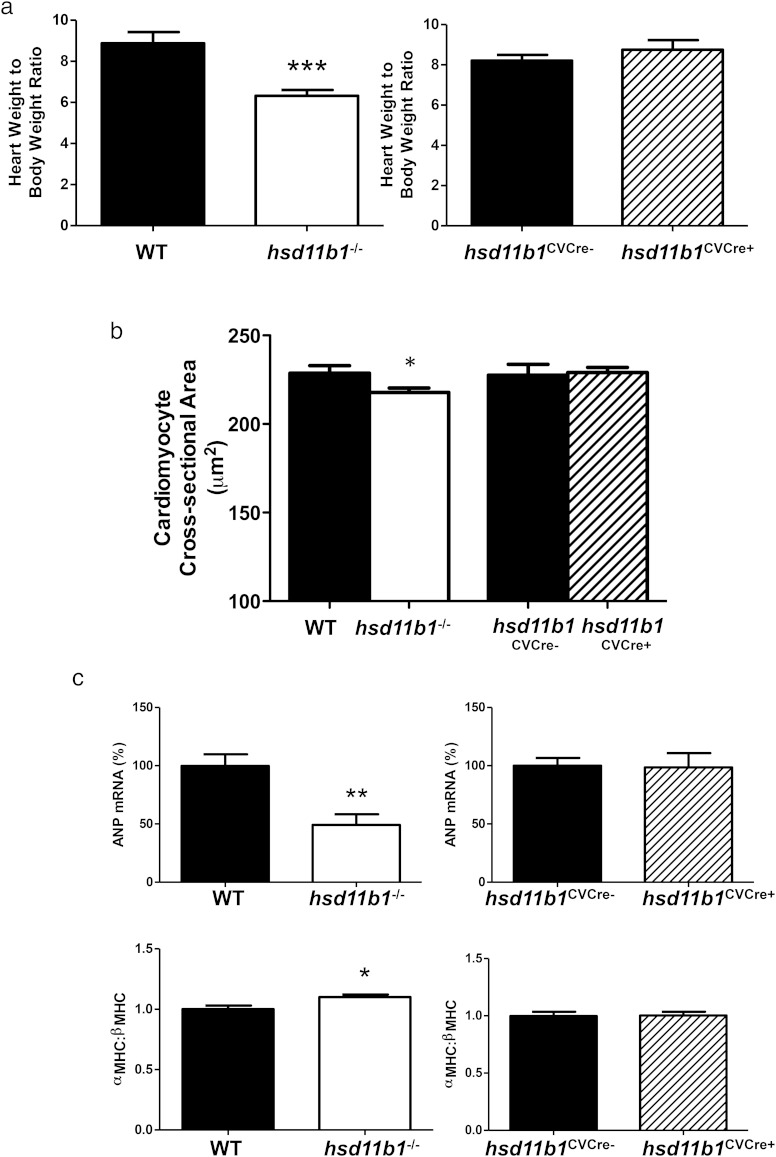
Hypertrophic remodeling and expression of fetal genes are inhibited in mice with global, but not cardiovascular, 11β-HSD1 deficiency. Heart weight to body weight ratio (A) and cardiomyocyte cross-sectional area (B) were compared in mice global 11β-HSD1-deficient mice (*Hsd11b1*^−/−^) and WT controls and in mice with cardiomyocyte and vascular smooth muscle-specific deletion (*Hsd11b1*^CVCre+^) compared with respective floxed control (*Hsd11b1*^CVCre−^) at 8 weeks after induction of MI by CAL. C, Expression of fetal genes ANP (*Nppa*) (upper panel) and α-MHC (*Myh6*), expressed relative to β-MHC (*Myh7*) (lower panel) was determined by real-time PCR. *, *P* < .05; **, *P* < .01; ***, *P* < .005 vs matched control; n = 6–8/group.

**Figure 5.**
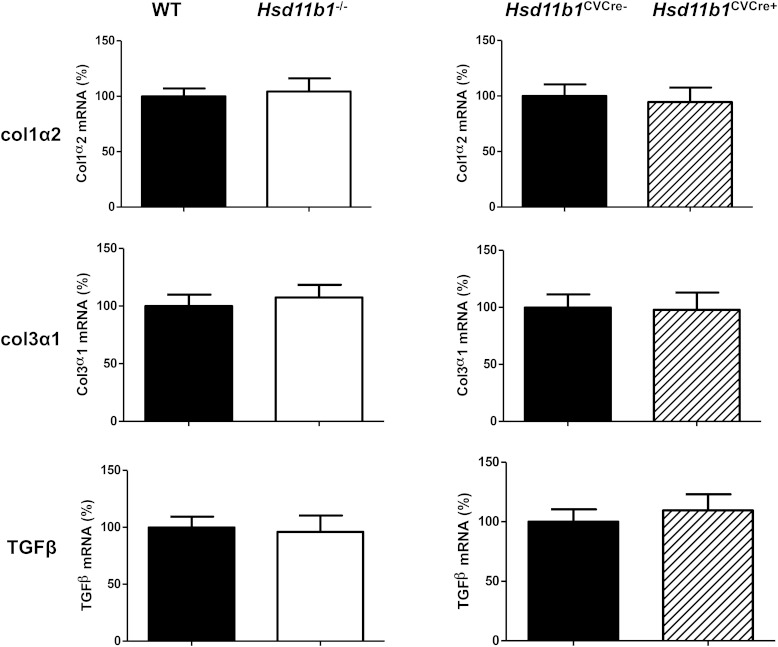
Fibrotic gene expression after MI is not influenced by 11β-HSD1. Myocardial *Col1a2* (upper panel), *Col3a1* (middle panel), and *Tgfb1* (lower panel) expression determined by qRT-PCR in hearts from WT mice, mice with global depletion of 11β-HSD1 (*Hsd11b1*^−/−^), mice with deletion in cardiac and vascular smooth muscle cells (*Hsd11b1*^CVCre+^), and floxed Cre-ve controls (*Hsd11b1*^CVCre−^); n = 6–8/group.

MRI analysis conducted at 8 weeks after induction of MI revealed a reduction in myocardial mass in *Hsd11b1*^−/−^ (120 ± 7.3 mg) compared with WT mice (160 ± 5.7 mg, *P* < .01), and this was confirmed by postmortem gravimetric analysis (*P* < .001) (Figure 4A). Reduced hypertrophic remodeling in hearts from *Hsd11b1*^−/−^ mice was evidenced by a significant decrease in cardiomyocyte cross-sectional area (*P* < .05) (Figure 4B) and in the expression of the fetal cardiomyocyte markers α-MHC (relative to β-MHC, *P* < .05) and ANP (*P* < .01) (Figure 4C), compared with their WT controls. Expression of the profibrotic genes *TGFB1*, and the *Col1a2* and *Col3a1* genes encoding the major collagens, was unaffected by global or cardiovascular-specific deficiency of 11β-HSD1 (Figure 5).

### Periinfarct angiogenesis and infarct expansion at 7 days after induction of MI are not influenced by cardiomyocyte and vascular smooth muscle selective depletion of *Hsd11b1*

**Figure 6.**
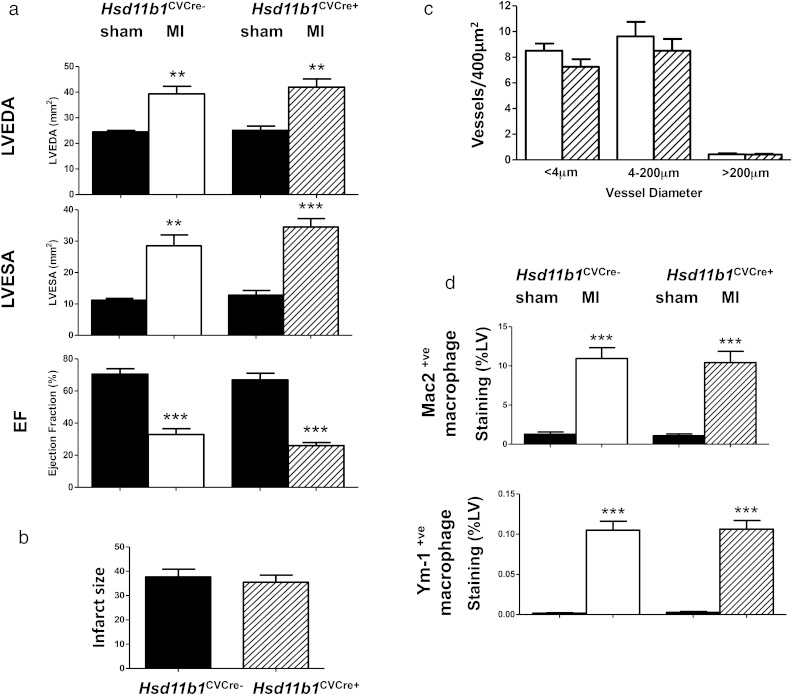
Cardiovascular selective deletion of 11β-HSD1 does not modify early infarct healing. Structural and functional remodeling (A), infarct size (B), periinfarct angiogenesis (C), macrophage density, and polarization (E) were all comparable in *Hsd11b1*^*CVCre*+*ve*^ and control floxed mice (*Hsd11b1*^*CVCre*+*ve*^) mice during infarct healing 7 days after induction of MI. LV end-diastolic area (LVEDA), LV end-systolic area (LVESA), fractional shortening (FS), and EF were assessed by high resolution ultrasound in mice 7 days after induction of MI by CAL or sham operation (sham). Macrophages were identified by immunoreactivity for the secreted protein Mac 2 (D, upper panel) and alternatively activated macrophages by expression of immunoreactive Ym-1 (D, lower panel). **, *P* < .01; ***, *P* < .005 vs sham of same genotype; n = 8/group.

As long-term outcomes after MI were not improved when *Hsd11b1* disruption was restricted to cardiomyocytes and vascular smooth muscle, a further study was conducted to investigate early wound healing and infarct expansion in these mice. By 7 days after induction of MI, during infarct repair, high resolution ultrasound showed that the extent of LV dilation and loss of function was similarly impaired in all mice relative to sham-operated mice (Figure 6A). Cardiomyocyte and vascular smooth muscle restricted deletion of *Hsd11b1* also failed to influence infarct size (Figure 6B) or the extent of periinfarct angiogenesis during infarct healing (Figure 6C). Macrophage recruitment was increased after MI relative to sham operation (Figure 6D), but neither recruitment nor polarization of macrophages towards a YM-1 positive “M2” macrophage phenotype was influenced in hearts from *Hsd11b1*^CVCre+^ compared with *Hsd11b1*^CVCre−^ mice (Figure 6D).

## Discussion

In this study, we present 3 important findings: 1) that 11βHSD1 amplifies infarct expansion and progression to heart failure after MI, supporting the use of pharmacological inhibitors of 11βHSD1 to prevent the development of heart failure in this setting; 2) that disruption of the *Hsd11b1* gene in cardiac muscle does not prevent the expression of fetal genes or hypertrophic remodeling after MI, suggesting that 11βHSD1 in cardiomyocytes does not play a direct role in regulating this process; and 3) that the promotion of periinfarct angiogenesis associated with prevention of early infarct expansion in mice with global 11βHSD1 deficiency (8) does not involve 11βHSD1 in smooth muscle cells of the vessel wall.

After MI, ventricular remodeling and heart failure develop in response to loss of contractile myocardial tissue that is not replaced after injury. The extent of this early injury is a key determinant of long-term outcome (28). In the present study, initial myocardial ischemic injury, as assessed by plasma levels of cardiac troponin I, was not influenced by 11β-HSD1 deficiency, confirming our previous observations where infarct injury was assessed directly early after MI (8). Glucocorticoids can protect the heart from ischemic injury, and systemic glucocorticoid, increased as a result of HPA activation in response to MI, is dominant in the first 24 hours after MI. We have already shown that this early peak in plasma glucocorticoid concentration after MI is not altered in 11β-HSD1-deficient mice; therefore, it is not surprising that early injury is also unaffected in these mice (8). However, by 8 weeks after induction of injury, the infarct had expanded and thinned to a lesser degree in mice with global deficiency of 11β-HSD1, and this was associated with less ventricular dilation, a key prognostic indicator of outcome in human heart failure (2, 29). Decreased dilation in the present study was accompanied by diminished expression of ANP, consistent with lowered wall stress (30), as well as reduction of ventricular dysfunction and pulmonary edema, together indicating prevention of progression to heart failure. This outcome is consistent with a detrimental influence of 11β-HSD1 on wound healing and remodeling that occur in response to injury.

Replacement of damaged myocardium by noncontractile scar tissue induces cardiomyocytes in the noninfarcted ventricle to undergo hypertrophic remodeling to maintain cardiac output, resulting in an increase in myocardial mass. Here, heart mass, assessed either in vivo by MRI, or ex vivo gravimetrically, was reduced in mice with global deficiency in 11β-HSD1. As well as increased cross-sectional area, cardiomyocytes undergoing pathological hypertrophy show reexpression of fetal genes, including those encoding ANP and β-MHC. Increased expression of β-MHC relative to the adult α-MHC isoform is associated with loss of cardiomyocyte contractility in murine models (31). In the present study it is clear that global deficiency in 11β-HSD1 suppresses hypertrophic remodeling and reduces reexpression of fetal genes consistent with retention of function and reduction of wall stress. Recent data have linked a common variation in the *Hsd11b1* gene to LV mass in man (32). Reversal of pathological hypertrophy by pharmacological inhibition of 11β-HSD1 in an MI-independent murine model also supports a specific role for 11β-HSD1 in the regulation of cardiomyocyte hypertrophy (19). However, neither modification of fetal genes, nor changes in myocyte cross-sectional area were observed when *Hsd11b1* deletion was limited to cardiomyocytes and vascular smooth muscle. This is consistent with a mechanism that is dependent on prohypertrophic signaling from other cells in the heart that express 11β-HSD1. Indeed, residual expression of *Hsd11b1*, evident in RNA and protein analysis of hearts from *Hsd11b1Cre*^+ve^ mice, supports sites of expression in the heart over and above cardiomyocytes and vascular smooth muscle. Transcriptomic analysis has revealed that *Hsdl11b1* is enriched 6-fold in cardiac fibroblasts relative to fibroblasts from other sources (33). Fibroblasts release a number of immunomodulatory and hypertrophy regulating factors and could be a key site for local regulation of cardiomyocyte growth during remodeling (34, 35). 11β-HSD1 has previously been shown to regulate the release of inflammatory mediators in synovial fibroblasts (36, 37), and this mechanism merits further investigation in the heart. Resident or recruited myeloid cells also express *Hsd11b1* and can regulate cardiomyocyte hypertrophy (38, 39). Alternatively, if infarct size is the primary driver for adaptive hypertrophy after MI (28), then reduction of infarct expansion in mice with global 11β-HSD1 deficiency may be sufficient to diminish changes in cardiomyocyte gene expression and size. It is feasible that loss of 11β-HSD1 in other organs, eg, the kidney, has indirect effects on hemodynamic stress during the development of heart failure.

We have previously demonstrated that periinfarct angiogenesis during infarct healing is enhanced in globally 11β-HSD1-deficient mice (8, 10) and that this is associated with development of shorter and thicker infarcts (8). *Hsd11b1* is expressed in the smooth muscle, but not the endothelium, of murine vasculature (17), where it regulates inflammation and neointimal proliferation (40, 41). It has been suggested that 11β-HSD1 in the vessel wall may contribute to regulation of angiogenesis (10). However, in the present study targeted deletion of 11β-HSD1 in vascular smooth muscle failed to recapitulate enhancement of periinfarct angiogenesis after MI. Enhancement of angiogenesis was previously linked to promotion of alternative macrophage activation during early infarct healing in globally *Hsd11b1*-deficient mice (8), this was also absent in mice with targeted deletion of *Hsd11b1* in cardiomyocytes and vascular smooth muscle. Again, other cells present in the heart, including fibroblasts and myeloid cells, express 11β-HSD1 and can release molecules that regulate inflammation and angiogenesis (36–38), and these sites may be important for the effects of global 11β-HSD1 deficiency on infarct healing after MI. Alternative macrophage activation can also promote fibrosis (42), potentially having a detrimental influence on cardiac function in the longer term. However, there was no evidence in the present study for an influence of 11β-HSD1 deficiency on the expression of a range of fibrosis associated genes in the failing LV.

The discussions above are focused around the principal that the primary function of 11β-HSD1 is to modulate intracellular availability of active glucocorticoid metabolites. Evidence is provided for appropriate reduction of gene expression and protein content in mice with targeted deletion of *Hsdl11b1*, but a limitation of the study is the lack of direct evidence for reduction of active glucocorticoid regeneration through 11β-HSD1 activity in the targeted cells. Although it is not therefore possible to state conclusively that reduced availability of locally generated glucocorticoids is key for the study outcomes, this limitation does not detract from the conclusion that 11β-HSD1 is a valid therapeutic target in the heart after MI.

A number of pharmacological inhibitors of 11β-HSD1 have been developed for the treatment of atherosclerotic and metabolic disease (15). We have found that 11β-HSD1 inhibitor can promote early angiogenesis and prevent infarct expansion when given immediately after MI (16), the present data suggests that pharmacological 11β-HSD1 inhibition also has the potential to reduce progression to heart failure. The use of mineralocorticoid receptor (MR) antagonists in patients with heart failure is now well established (43). Recent clinical and experimental studies have highlighted additional benefits of MR antagonists when given early after MI, which include promotion of angiogenesis and prevention of infarct expansion (44, 45). Studies in mice with cardiomyocyte-restricted inactivation of the MR gene suggest that the clinical benefits of MR blocking therapy in MI and heart failure are mediated largely via cardiomyocyte-dependent mechanisms (46). As the present data show that the effects of *Hsd11b1* gene targeting are cardiomyocyte independent, 11β-HSD1 inhibitors may offer additional benefits in patients already receiving MR antagonist therapy or an alternative in patients for whom hyperkalemia precludes the use of MR antagonists (47).
